# Regional Arterial Infusion with Lipoxin A_4_ Attenuates Experimental Severe Acute Pancreatitis

**DOI:** 10.1371/journal.pone.0108525

**Published:** 2014-09-29

**Authors:** Fajing Yang, Jianming Xie, Weiming Wang, Yangyun Xie, Hongwei Sun, Yuepeng Jin, Dan Xu, Bicheng Chen, Roland Andersson, Mengtao Zhou

**Affiliations:** 1 Department of Surgery, The First Affiliated Hospital, Wenzhou Medical University, Wenzhou, Zhejiang Province, China; 2 School of Optometry and Ophthalmology, Wenzhou Medical University, Wenzhou, Zhejiang Province, China; 3 Zhejiang Provincial Top Key Discipline in surgery, Wenzhou Key Laboratory of Surgery, Department of Surgery, The First Affiliated Hospital, Wenzhou Medical University, Wenzhou, Zhejaing Province, China; 4 Department of Surgery, Clinical Sciences Lund, Lund University and Lund University Hospital, Lund, Sweden; Kaohsiung Medical University Hospital, Kaohsiung Medical University, Taiwan

## Abstract

**Objective:**

Investigate the therapeutic effect of regional arterial infusion (RAI) with Aspirin-Triggered Lipoxin A_4_ (ATL) in experimental severe acute pancreatitis (SAP) in rats.

**Materials and Methods:**

SAP was induced by injection of 5% sodium taurocholate into the pancreatic duct. Rats with SAP were treated with ATL (the ATL group) or physiological saline (the SAP group) infused via the left gastric artery 30 min after injection of sodium taurocholate. The sham group was subjected to the same surgical procedure, though without induction of SAP. Serum levels of amylase, phospholipase A2 (PLA2), interleukin-1β (IL-1β), IL-6 and tumor necrosis factor-α (TNF-α) were measured at 12 and 24 h after induction of SAP. Ascitic fluid, the pancreatic index (wet weight ratio) and myeloperoxidase (MPO) levels in the pancreas were determined and histopathological findings were evaluated. The expression of intercellular adhesion molecule-1 (ICAM-1), platelet endothelial cell adhesion molecule-1 (PECAM-1), NF-κB p65, and heme oxygenase-1 (HO-1) in the pancreas were estimated by immunofluorescence and western blot, respectively.

**Results:**

ATL rats had lower serum levels of TNF-α, IL-1β, and IL-6 (*P*<0.01), PLA_2_ (*P*<0.05), and amylase levels (*P*<0.05) studied as compared with the SAP group. The pancreatic index in the ATL group decreased only at 24 h as compared with the SAP group (*P*<0.05). The histopathological findings and MPO levels in the pancreas significantly decreased in the ATL group as compared to the SAP group (*P*<0.05 and *P*<0.01, respectively). Immunofluorescence and western blot showed that ATL attenuated the expression of NF-κB p65, ICAM-1 and PECAM-1 in the pancreas, and increased the expression of HO-1 in SAP animals.

**Conclusions:**

We demonstrated that RAI with ATL attenuated the severity of experimental SAP, maybe achieved by improving the expression of HO-1, and down-regulating the NF-κB signaling pathway, with decreased expression of ICAM-1 and PECAM-1 and reduced generation of pro-inflammatory cytokines.

## Introduction

The importance of inflammatory cells, their activations, and the inflammatory cascades involved in severe pancreatitis (SAP) has been emphasized [1]. The main factor regulating the concomitant course of disease during the early phase of SAP is the magnitude of the systemic inflammatory response syndrome (SIRS). SIRS is the main driving factor for the development of e.g. the acute respiratory distress syndrome (ARDS), multiple organ dysfunction syndrome (MODS) and the main cause of death [2]–[5]. Proinflammatory cytokines have been considered responsible for systemic manifestations and complications of SIRS in SAP [6]. Cytokines cause both local and distant upregulation of adhesion molecules that further trigger inflammatory cascades by enhancing leukocyte migration, complement activation, neutrophil degranulation, production of phospholipase A2 (PLA2), nitric oxide, and oxygen radicals [7]. Minimizing the inflammatory cytokine response in order to avoid excessive SIRS thus seems essential.

Lipid-derived lipoxins are produced at the site of vascular and mucosal inflammation and possess powerful anti-inflammatory properties [8]. Lipoxin A_4_ represents a key member among lipoxins, [9] and during the late phase of inflammation it prevents against further inflammatory response by influencing neutrophil apoptosis [10], inhibiting neutrophil chemotaxis and respiration [11], and stimulate the uptake and clearance of apoptotic polymorphonuclear neutrophils [12]. ATL, as the isomer of Lipoxin A_4_, is derived from the aspirin-triggered formation of 15(R)-HETE from arachidonic acid [13], [14]. ATL inhibits LTB_4_-induced chemotaxis, adherence, and transmigration of neutrophils with twice the potency of Lipoxin A_4_ activity in the nM range [14], [15], and it is furthermore resistant to metabolic inactivation [16].

ATL can reduce the excretion of proinflammatory cytokines and modulate excessive neutrophil stimulation, factors that also are involved in the development of both local and systemic complications of SAP. In a previous study, we demonstrated that administration of Lipoxin A_4_ attenuated the severity of acute pancreatitis and SIRS during the early phase of SAP in a rat model [17], but we also found a dose-dependent in the therapeutic effect of Lipoxin A_4_, rendering Lipoxin A_4_ too expensive for further studies. Continuous regional arterial infusion (CRAI) has been reported as an effective route for drug delivery also in acute pancreatitis [18], [19], enhancing the therapeutic effect by increasing drug concentrations in the pancreas with minimal systemic toxic side effects. We have demonstrated that drug concentrations in the pancreas increased significantly through CRAI as compared to peripheral venous administration [20]. The aim of the present study was to investigate the therapeutic effect and potential mechanisms of CRAI with ATL during the early phase of experimental SAP.

## Materials and Methods

### Experimental animals

A total of 42 healthy male Sprague-Dawley rats, weighing 220–280 g, were used, supplied by the Laboratory Animal Centre of Wenzhou Medical College (Wenzhou, China). The experimental protocol was approved by the Institutional Animal Committee of Wenzhou Medical College (document number: wydw2012–0026). All animals received care in accordance with ‘Guide for the Care and Use of Laboratory Animals’. All rats were housed under diurnal lighting condition (12/12 h light/dark cycle with humidity of 60±5%, 22±3°C) and fed a standard rat chow and water except for a day of fasting before the operation.

### Induction of severe acute pancreatitis and cannulation to the celiac artery

The SAP rat model was established according to what has been described by Aho et al [21]. Briefly, the rats were operated under aseptic conditions, using 10% chloral hydrate (0.3 ml/100 g) anesthesia injected intraperitoneally. The pancreas was exteriorized through a midline abdominal incision, the proximal bile duct was clamped at the level of the distal bile duct, and the distal pancreatic duct was cannulated using a 5-gauge scalp needle through the duodenal wall. SAP was induced by the intraductal injection of 5% sodium taurocholate (0.1 ml/100 g) using a microinjection pump at a speed of 0.1 ml/min. After induction of SAP and exposing the left gastric artery, a PE-10 catheter (AniLab Software and Instruments Co. Ltd. Ningbo, China) was inserted in a retrograde direction and the tip of the catheter was placed at the celiac artery. After injection of methylene blue, we confirmed that the left and right lobes of the pancreas were stained.

### Experimental design

All animals were randomly divided into three groups (fourteen rats in each group): 1. The ATL group with induction of SAP administration of ATL (0.1 mg/kg; Cayman Chemical Company, Ann Arbor, Michigan, USA) via the left gastric artery 30 min after TAP induction; 2. The SAP group administered an equal amount of physiological saline via the left gastric artery after the induction of SAP; and 3. The sham group subjected to a similar surgical procedure, though without induction of SAP, and administration of an equal amount of physiological saline via the left gastric artery after 30 min.

### Specimen collection

Seven rats from each group were sacrificed under anesthesia at 12 and 24 h, respectively, after the induction of SAP. Ascitic fluid, blood, and pancreatic tissue were collected at these time points for subsequent analysis.

### Weight of ascites and wet weight ratio of the pancreas

Ascitic fluid was collected with cotton balls and the weight of the whole wet pancreas was measured.

The pancreatic index refers to the percentage of the pancreatic wet weight as compared to the body weight [22]. This ratio (pancreatic weight g/body weight g×1,000) was utilized to evaluate the degree of pancreatic edema.

### Histopathological analysis

Specimens of the pancreas was harvested and fixed in 10% formaldehyde solution for 48 h, dehydrated in graded alcohol series, cleared with xylene and embedded in paraffin. Tissue sections (4 µm) were stained with hematoxylin-eosin (H&E) for general morphology and observed under a Leica fluorescent microscope (Leica Microsystems, Wetzlar, Germany). The pathological grading described by Schmidt et al [23] takes all factors, including pancreatic edema, acinar necrosis, hemorrhage and fat necrosis, as well as inflammation and perivascular infiltration, into consideration when scoring the pancreatic injury in experimental SAP [23]. The mean value of the total score of these four parameters in each group was analyzed. The pathological sections were examined by two experienced pathologists from the Department of Pathology, Wenzhou Medical College, in a blinded fashion.

### Biochemical analysis

Serum amylase activity was determined by means of iodine-amylum colorimetry (Hitachi 917 autoanalyzer, Tokyo, Japan) and expressed in units per liter. Serum levels of PLA_2_, as well as serum levels of TNF-α, IL-1β and IL-6, were quantified according to the manufacturer’s instructions and guidelines using enzyme-linked immunosorbent assay (ELISA) kits (Shanghai Xitang Biotechnology Co., Ltd, Shanghai, China or R&D Systems, Inc, Minneapolis, MN, USA) and expressed respectively in nanograms per milliliter or in picograms per milliliter.

The MPO activity in pancreatic tissue was determined according to the manufacturer’s instructions and guidelines using MPO testing kit (Nanjing Jiancheng Bioengineering Institute, Nanjing, China) and expressed in units per gram.

### Immunofluorescence analysis

Briefly, paraffin-embedded pancreatic tissue sections (4 µm) were rehydrated first in xylene and then in graded ethanol solutions. For antigen retrieval, the slides were incubated in citrate buffer (PH 6.0) in a microwave oven. After being allowed to cool to room temperature and rinsed with phosphate buffered saline (PBS), the sections were dealt with 0.3% H_2_O_2_ for 10 minutes to block endogenous peroxidase activity. Non-specific binding was blocked with 10% goat serum albumin in PBS for 30 minutes at 37°C. Then, the slides were incubated overnight at 4°C in a humidified chamber with rabbit polyclonal antibody anti-NF-κB p65 (1∶200, Bioworld Technology Inc, Louis Park, MN, USA ), anti-ICAM-1 (1∶200, Bioworld Technology Inc), anti-PECAM-1 (1∶200, Santa Cruz Biotechnology, Santa Cruz, CA, USA) and mouse polyclonal antibody anti-HO-1 (1∶100, Abcam Inc, Cambridge, MA, USA). The second day, the sections were washed with PBS (5 min×3) and incubated with goat anti-rabbit fluorescence isothiocyanate (FITC) conjugated secondary antibody (1∶50, Beyotime Technology Inc, Jiangsu, China) or goat anti-mouse tetramethylrhodamine isothiocyanate (TRITC) conjugated secondary antibody (1∶50, ZSJQ-BIO, Bejing, China) in dark for 2 h at room temperature. The sections were then washed with PBS (5 min×3) and incubated with the nucleus specific counterstain 4,6-diamidino-2-phenylindole dihydrochloride (DAPI) to highlight cell nuclei. The slides were visualized under a fluorescent microscope (Leica Microsystems) using Excitation wavelength/Emission wavelength of 358 nm/460 nm for DAPI, 492 nm/520 nm for FITC, and 550 nm/580 nm for TRITC. For evaluation of ICAM-1 and PECAM-1 expressions, ten random fields across each section were selected for analysis of mean fluorescence intensity. For evaluation of NF-κB p65 and HO-1 expressions, ten random fields across each section were selected for analysis of the positively expressed cells of per 0.245 mm^2^.

### Western blot

The total protein and nuclear proteins were extracted, respectively, from rat pancreatic tissue following the manufacturer’s protocols (Beyotime Technology Inc, Jiangsu, China) at 12 and 24 h after induction of SAP. Protein concentration was determined in the supernatant using a BCA Protein Assay Reagent Kit (Beyotime Technology Inc). For each lane, an equivalent amount of 40 µg total protein samples, as well as 20 µg nuclear protein samples were dissolved, respectively, in 20 µl of 1 × sodium dodecyl sulfate loading dye and boiled for 5 minutes. The two kind of protein samples were separated by a 10% sodium dodecyl sulfate-polyacrylamide gel and transferred 1 h onto PVDF membranes (Solarbio Technology Inc, Bejing, China) via electroblotting (Wet Trans-blot; Bio-Rad, Hercules, CA, USA). The membranes were blocked for 2 h at room temperature with a blocking solution (5% nonfat milk in Tris buffered saline with Tween 20 (TBST)) and incubated overnight at 4°C with rabbit polyclonal antibody anti-NF-κB p65 (1∶500, Bioworld Technology Inc), anti-ICAM-1 (1∶500, Bioworld Technology Inc), anti-PECAM-1 (1∶500, Santa Cruz Biotechnology), anti-GAPDH (1∶2500, Bioworld Technology Inc), anti-Lamin B2 (1∶1000, Bioworld Technology Inc) and mouse polyclonal antibody anti-HO-1 (1∶250, Abcam Inc). After 3 washing steps with TBST (10 min×3), the membranes were incubated with secondary antibodies (1∶2500, Beyotime Technology Inc) diluted with 5% nonfat milk in TAST for 2 h at room temperature. Then, the membranes were washed again with TBST (10 min×3) and the immunoreactive proteins were visualized with the use of ECL detection kit (Advansta, Menlo Park, California, USA) according to the manufacture’s instructions and exposed to a radiograph film (Yuehua Medical Instrument Factory Co., Ltd, Guangdong, China). The results of the graphs were analyzed by the Quantity One Version 4.6.2 Image software (Bio-Rad Laboratories).

### Statistical analysis

The data for seven rats in each group were analyzed by SPSS software (19.0 version) and expressed as mean ± SD. The statistical analysis was performed using a one-way analysis of variance (ANOVA) and multiple comparisons were analyzed by SNK-q test (when the variance was even) or Dunnett’s T3 test (when the variance was uneven). A Kruskal-Wallis H test was used to analyze the differences in pathological score and multiple comparisons were evaluated with Nemenyi test. For all analyses, statistical significance was defined as *P*<0.05.

## Results

### Ascites, serum amylase activity and serum levels of PLA2

**Figure 1:**
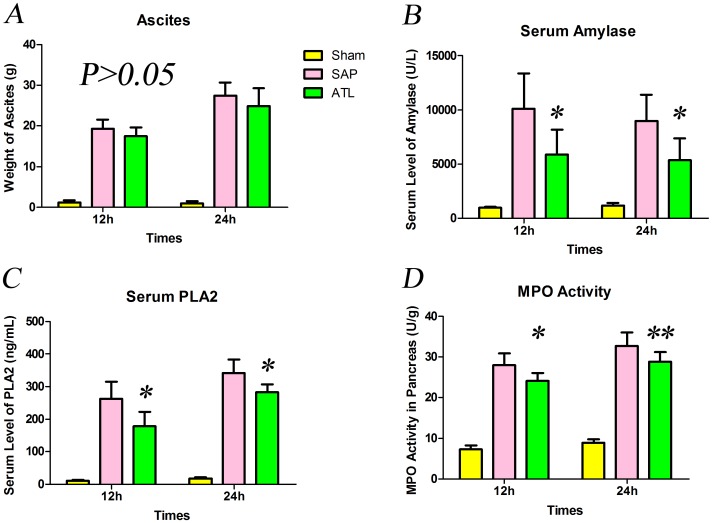
The weight of ascetic, serum levels of amylase and PLA_2_, and MPO activity in pancreas. **A)** The weight of ascitic fluid increased after induction of severe acute pancreatitis (SAP), though without significant difference after regional arterial infusion (RAI) with Asprin-Triggered Lipoxin A_4_ (ATL). **B)** The amylase activity increased rapidly in the SAP group both at 12 and 24 h compared to the sham group and decreased significantly after RAI with ATL (*P*<0.05). **C)** Serum levels of PLA_2_ (ELISA) increased in the SAP group vs. the sham group, higher at 24 h than at 12 h both in the SAP and ATL groups. PLA_2_ serum levels in the ATL group decreased (*P*<0.05 vs. SAP). **D)** MPO levels in the pancreas increased after SAP induction, significantly attenuated after RAI with ATL both at 12 and 24 h (*P*<0.05 and *P*<0.01, respectively). Seven rats were studied in each experimental group at each time point. The results are expressed as means and SD. * *P*<0.05 and ** *P*<0.01, ATL group vs. the SAP group.

Abundance of hemorrhagic ascites was observed in the abdominal cavity both 12 and 24 h after induction of SAP. The weight of ascitic fluid increased in the SAP and ATL groups, progressing over time, though there were no significant changes between the SAP and ATL groups both at either 12 or 24 h (*P*>0.05; Fig. 1A).

The SAP model was characterized by substantially increased serum levels of amylase and PLA_2_. Serum levels of amylase and PLA_2_ were normal in the sham group, but increased after induction of SAP. PLA_2_ gradually increased both in the SAP and ATL groups within 24 h, while amylase decreased at 24 h after induction of SAP, both in the SAP and ATL groups, compared to the 12 h time point. Serum levels of amylase and PLA_2_ significantly decreased in the ATL group both at 12 and 24 h as compared to the SAP group (*P*<0.05; Fig. 1B and C).

### Pancreatic index and MPO activity

The pancreatic index is described in Table 1. A significant decrease in the ATL group was found at 24 h.

**Table 1:** The scores of pathological grading of pancreatic injury and pancreatic index.

Time point	Sham group		SAP group		ATL group		*P* value	
	Pathologic grade	Pancreatic index	Pathologic grade	Pancreatic index	Pathologic grade	Pancreatic index	Pathologic grade	Pancreatic index
12 h	0.54±0.14	4.39±0.29	11.49±1.15	6.59±0.52	9.19±1.23	9.59±1.33	<0.05	>0.05
24 h	0.58±0.13	4.40±0.25	12.11±1.30	6.85±0.42	9.59±1.33	6.24±0.49	<0.05	<0.05

SAP = severe acute pancreatitis.

ATL = SAP+regional arterial infusion ATL.

*P* value = the statistical value of the SAP vs. the ATL group.

To evaluate polymorphonuclear leukocyte (PMN) infiltration into the pancreas, MPO activity was measured in tissue samples. MPO activity was minimal in the sham group, whereas being clearly detectable in the SAP group. The ATL group had a significant reduction in pancreatic MPO activity both at 12 h (*P*<0.05) and 24 h (*P*<0.01) as compared to the SAP group (Fig. 1D).

### Histopathological examination

**Figure 2:**
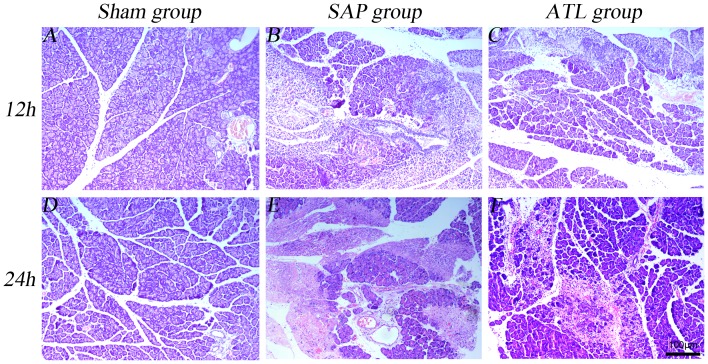
Representative photographs of HE stained pancreas. Pathological section of pancreatic tissue under light microscope (HE staining × 100). The sham group (A and D) was normal. Tissue edema, inflammatory cell infiltration, hemorrhage, and necrosis in the pancreas were observed in the severe acute pancreatitis (SAP) group (B and E). Changes were substantially ameliorated by regional arterial infusion (RAI) with Asprin-Triggered Lipoxin A_4_ (ATL) (Fig. C and F).

The induction of SAP was successful in all animals. Patchy putrescent changes with saponified spots and color alterations was seen in the pancreatic tissue and hemorrhagic ascites were found in the abdominal cavity both at 12 and 24 h after induction of AP. Microscopically, pancreatic edema, acinar necrosis, hemorrhage and fat necrosis, as well as inflammation and perivascular infiltration in the pancreas were observed both in the SAP and ATL groups (Fig. 2). As mentioned, the histopathological examination of the pancreas was scored according to the method reported by Schmidt et al [23]. The pathological grade gradually increased in the SAP and the ATL groups (both at 12 and 24 h), though the pathological scores were significantly lower in the ATL as compared to the SAP group (*p*<0.05; Table 1).

### Serum levels of proinflammatory cytokines

**Figure 3:**
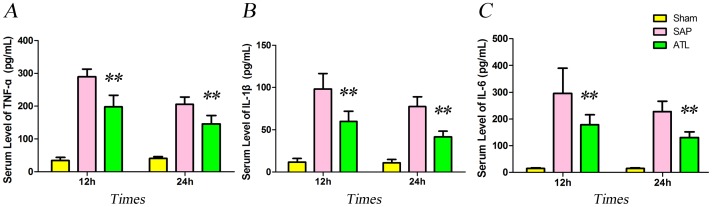
The serum levels of pro-inflammatory cytokines. Increases in interleukin-1β (IL-1β), IL-6, and tumor necrosis factor-α (TNF-α) generation at 12 and 24 h after induction of severe acute pancreatitis (SAP). These increases in cytokine levels were significantly ameliorated by regional arterial infusion (RAI) with Asprin-Triggered Lipoxin A_4_ (ATL) (*P*<0.01). Seven rats were studied in each experimental group at each time point. The results are expressed as means and SD. * *P*<0.05 and ** *P*<0.01, ATL vs. the SAP group.

Serum levels of TNF-α, IL-1β and IL-6 were negligible in the sham group, while they increased after induction of SAP. Serum proinflammatory cytokine levels of TNF-α, IL-1β and IL-6 presented a declining tendency at 24 h both in the SAP and ATL groups as compared with the levels at 12 h. In the ATL group, they decreased both at the 12 and 24 h time points as compared with the SAP group (*P*<0.01; Fig. 3).

### Analysis of HO-1 and NF-κB p65 Expression

**Figure 4:**
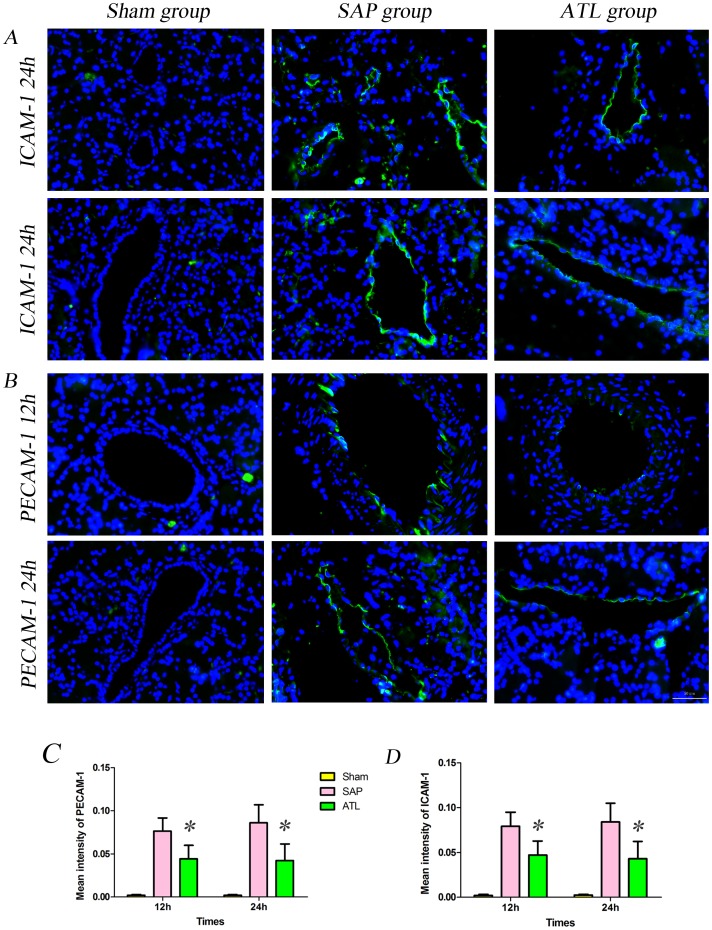
Immunofluorescence staining for ICAM-1 and PECAM-1 in the pancreas. **A)** The expression of ICAM-1 in vascular endothelial cells in the pancreas increased after induction of severe acute pancreatitis (SAP) compared to the sham group; findings significantly attenuated by regional arterial infusion (RAI) with Asprin-Triggered Lipoxin A_4_ (ATL). **C)** Fluorescence intensity for ICAM-1. The results are expressed as means and SD. The mean fluorescence intensity of ICAM-1 decreased significantly in the ATL group both at 12 and 24 h (*P*<0.05 vs. SAP). **B)** The expression of PECAM-1 in vascular endothelial cells in the pancreas increased significantly after induction of SAP compared to the sham group, attenuated by RAI with ATL. **D)** Bar graph demonstrating fluorescence intensity for PECAM-1. The results are expressed as means and SD. The mean fluorescence intensity of PECAM-1 decreased significantly in the ATL group both at 12 and 24 h (*P*<0.05). * *P*<0.05 and ** *P*<0.01, ATL vs. the SAP group.

To determine whether the proteins of HO-1, NF-κB p65, ICAM-1 and PECAM-1 in the pancreas were involved in experimental SAP, their expressions were assessed by immunofluorescence and western blot. Figure 4 demonstrate immunofluorescence staining of HO-1 and NF-κB p65, counterstained with DAPI. Increased positive expressions of HO-1 (Fig. 4A) and NF-κB p65 (Fig. 4B) were demonstrable in the pancreas in the SAP group as compared to the sham group. The labeled HO-1 protein was mostly observed in the cytoplasm, while the positive expression of NF-κB p65 was not only detected in the cytoplasm, but also vastly expressed in the nucleus after SAP induction. The expression of HO-1 continuously increased in the ATL group as compared to the SAP group at 12 and 24 h, respectively, while the expression of NF-κB p65 was obviously attenuated. Quantification of the HO-1 and NF-κB p65 results are depicted in Fig. 4C (*P*<0.05) and Fig. 4D (P<0.05 at 12 h; P<0.01 at 24 h), respectively, quantified per 0.245 mm^2^ of positively expressed cells. Western blot also showed that the expression of HO-1 in the pancreas significantly increased at a protein level (*P*<0.01; Fig. 6A and E) and the expression of NF-κB p65 in the nucleus decreased (*P*<0.05 at 12 h; *P*<0.01 at 24 h; Fig. 6B and F) in the ATL group as compared to the SAP group.

### Analysis of ICAM-1 and PECAM-1 expression

**Figure 5:**
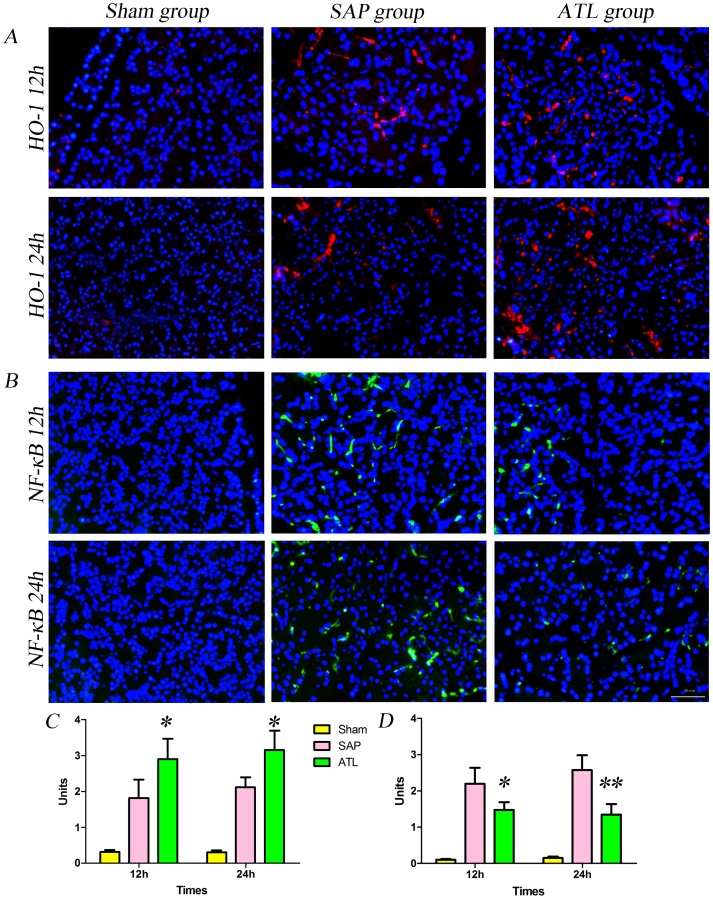
Immunofluorescence staining for HO-1 and NF-κB p65 in the pancreas. **A)** The expression of heme oxygenase-1 (HO-1) in the pancreas increased after induction of severe acute pancreatitis (SAP) compared to the sham group. After regional arterial infusion (RAI) with Asprin-Triggered Lipoxin A_4_ (ATL), the expression of HO-1 increased significantly compared to the SAP group. **C)** Bar graph demonstrating positively expressed cells of HO-1 per 0.245 mm^2^ (results are described as means and SD). The positively expressed cells of HO-1 per 0.245 mm^2^ increased significantly in the ATL group both at 12 and 24 h (*P*<0.05 vs. SAP). **B)** The expression of nuclear NF-κB in the pancreas increased significantly after induction of SAP compared to the sham group, attenuated by RAI with ATL. **D)** Bar graph demonstrating the positively expressed cells of NF-κB per 0.245 mm^2^ (results are expressed as means and SD). The positively expressed cell of NF-κB per 0.245 mm^2^ decreased in the ATL group both at 12 and 24 h (*P*<0.05 or *P*<0.01 vs. SAP). * *P*<0.05 and ** *P*<0.01, ATL vs. the SAP group.

**Figure 6:**
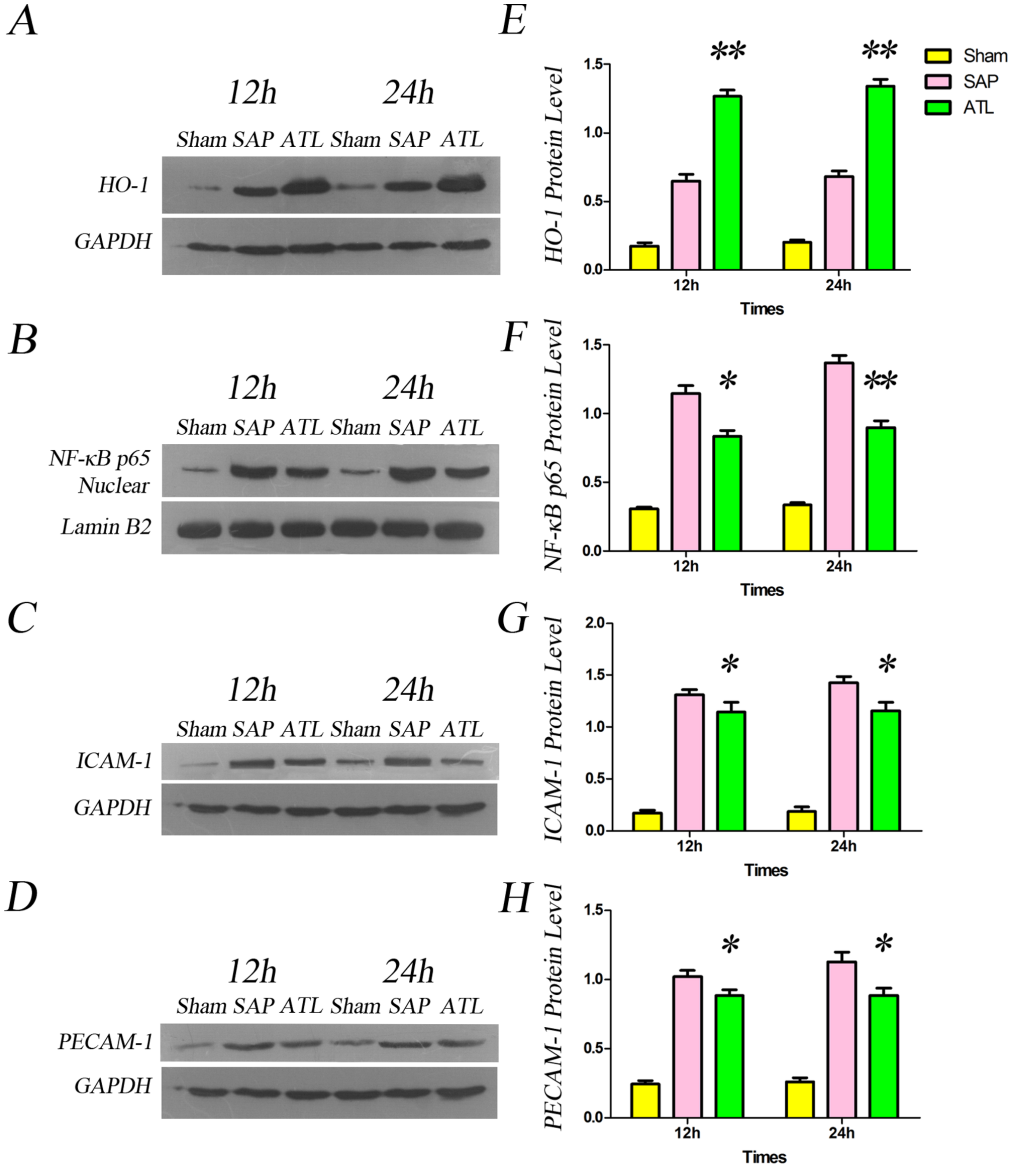
Western blot analysis of heme oxygenase-1 (HO-1), NF-κB p65, ICAM-1 and PECAM-1 in the pancreas. **A)** Representative photographs of HO-1 protein levels in the pancreas in the three groups. **E)** Bar graph quantifying the HO-1 protein level in the pancreas. HO-1 protein levels in the pancreas increased after induction of severe acute pancreatitis (SAP) compared to the sham group, significantly increased following regional arterial infusion with Asprin-Triggered Lipoxin A_4_ (ATL) (*P*<0.01 vs. SAP). **B)** Representative photographs of nuclear NF-κB protein level in the pancreas in the three groups. **F)** Bar graph quantifying nuclear NF-κB protein levels in the pancreas. Protein levels of nuclear NF-κB in the pancreas increased after induction of SAP compared to the sham group, and decreased significantly in the ATL group both at 12 and 24 h (*P*<0.05 or *P*<0.01, respectively, vs. SAP). **C)** Representative photographs of ICAM-1 protein levels in the pancreas in the three groups. **G)** Bar graph quantifying ICAM-1 protein levels in the pancreas, increased after induction of SAP as compared to the sham group, and significantly decreased in the ATL group (*P*<0.05 vs. SAP). **D)** Representative photographs of PECAM-1 protein levels in the pancreas in the three groups. **H)** Bar graph quantifying PECAM-1 protein levels in the pancreas, increased after induction of SAP (vs. sham) and significantly decreased in the ATL group both at 12 and 24 h (*P*<0.05 vs. SAP). The results are expressed as means and SD. * *P*<0.05 and ** *P*<0.01, ATL vs. the SAP group.

Figure 5 demonstrate immunofluorescence staining of ICAM-1 and PECAM-1, counterstained with DAPI. Limited labeled ICAM-1 and PECAM-1 expression were detected in microvascular endothelial cells (ECs) in the pancreas in the sham group, while the expression was increased after SAP induction. The expression of ICAM-1 and PECAM-1 significantly increased in the ATL group as compared with the SAP group, both at 12 and 24 h. Quantification of ICAM-1 and PECAM-1 are presented in Fig. 5C (*P*<0.05) and Fig. 5D (*P*<0.05), quantified by mean inmunofluoresence intensity. Consistent with the results of immunofluorescence, western blot demonstrated that the expression of ICAM-1 and PECAM-1 proteins in the pancreas significantly decreased in the ATL group at both time points as compared to the SAP group (*P*<0.05; Fig. 6).

## Discussion

We have previously investigated the effect of intravenous administration of Lipoxin in severe acute pancreatitis (SAP) in a rat model [17]. The effectiveness of Lipoxin administration through regional arterial infusion (RAI) in experimental SAP has though never been studied. Continuous regional arterial infusion (CRAI) per se in the treatment of SAP had been utilized for both basic research and clinical application in our department since the 1990 s [24]. Japanese studies have also documented the effectiveness of CRAI approach, reducing mortality in patients with SAP [25], [26], which implies that intra-arterial infusion increase in local drug concentrations in pancreatic tissue as compared to corresponding intravenous injections [19], [27]. In the present study, RAI with ATL attenuated the severity in a SAP model and down-regulatea SAP-associated SIRS. ATL possess dual anti-inflammatory and pro-resolution activities, demonstrated in multiple acute and chronic inflammatory conditions [28].

The magnitude of pancreatitis, including edema, infiltration, hemorrhage, and necrosis of pancreatic tissue, was graded according to the method reported by Schmidt et al [23], with objective evaluation criteria determining the severity of SAP. Microscopically, a decreased degree of pancreatic edema, leukocyte infiltration and pancreatic damage was seen after RAI with ATL. Tissue MPO activity is predominantly found in the azurophilic granules of PMN and utilized to estimate tissue PMN accumulation in inflamed tissues and correlates with the number of PMNs determined histochemically [29]. PLA_2_, an enzyme involved in lipid metabolism, hydrolyzes phospholipids to liberate arachidonic acid and lysophospholipids [30] and the latter has a cytotoxic function, causing acinar cell necrosis [31]. PLA_2_ seems to substantially contribute to the systemic inflammatory reaction in acute pancreatitis [32], and the serum catalytic activity of PLA_2_ is closely associated with the severity of pancreatitis [33]–[35]. In the present study, RAI with ATL reduced the pancreatic index, the MPO activity in the pancreas, as well as serum PLA_2_ activity, decreasing the degree of pancreatic edema, PMN infiltration, and acinar cell necrosis, overall suppressing the local pancreatic inflammation. Serum amylase is released from injured acinar cells in pancreatitis and the decreasing levels of serum amylase, thus implying less acinar cell damage.

ATL is involved in the resolution of inflammation. Previous studies have demonstrated that ATL can inhibit both monocytes and neutrophils from secreting IL-1 and TNF-α [36]. In the present pancreatitis study, RAI with ATL significantly decreased the content of pro-inflammatory cytokines, such as TNF-α, IL-1β and IL-6. TNF-α, a primary pro-inflammatory factor in the pathogenesis of SAP [37], directly injures cells of multiple organs, and causes ischemia, hemorrhage, necrosis, inflammation and edema. TNF-α also initiates a cascade reaction, accumulates neutrophils, increases ICAM and VCAM levels, and stimulates the production of NO, ROS and other pro-inflammatory factors, such as IL-6 and IL-1β [38]. IL-1 and TNF-α act both locally to aggravate the pancreatitis process and systemically, activating cytokines and leukocytes [17]. IL-6 has extensive pro-inflammatory effects causing tissue damage [39]. Levels of IL-1 and -6 have been reported as predictors of severity during the early phase of acute pancreatitis [40], [41]. Furthermore, a marked attenuation of the severity of SAP is observed when TNF-α and IL-1β receptors are blocked and mortality is reduced in TNF-α and IL-1β knockout mice subjected to SAP [42], [43]. Obviously, the loss of control of inflammatory cytokines during acute pancreatitis seems to be a key event, triggering cascade reactions causing massive necrosis of pancreatic tissue, complicated by multiple organ dysfunction. We found that ATL efficiently and early on inhibited cytokine release, attenuated local inflammatory response, decreased acinar cell injury, and modulated SIRS.

Adhesion molecules like ICAM-1 and PECAM-1 are scarcely expressed in endothelial cells, but increase in acute pancreatitis. PMN extravasation requires selectin-mediated tethering, ICAM-1-dependent firm adhesion, and PECAM-1-mediated transendothelial migration. PMNs transmigration from microvessels first requires firm adhesion to endothelial cells (ECs) mediated by ICAM-1 [44]. PECAM-1 expression in the EC plasma membrane is also critically involved in this process [45]. By using monoclonal ICAM-1 antibodies, in vitro PMN transmigration is significantly reduced, and the combination with monoclonal PECAM-1 antibodies renders a synergetic effect [46]. The increasing expressions of ICAM-1 and PECAM-1 in microvascular ECs thus represent essential steps for PMN transmigration. In the present study, the decreased expression of ICAM-1 and PECAM-1 following RAI with ATL reduced microvascular PMN infiltration in pancreatic tissue, and ameliorated the local inflammatory response, evidenced by a decrease in MPO activity in the pancreas. The decreased PMN transmigration further reduce the pro-inflammatory cytokine generation, decrease acinar cell injury, and attenuate SIRS.

ALT might beside attenuating the inflammation also possess potential cytoprotective properties. HO-1 plays an essential role in the anti-inflammatory and pro-resolution effects of ATL [47], [48]. HO-1 is a heat shock protein and a rate-limiting enzyme in the catabolism of heme to yield equimolar amounts of biliverdin, free iron, and carbon monoxide, with potent anti-inflammatory and cytoprotective functions [49]. Several studies have shown that HO-1 and its product, carbon monoxide, can reduce tissue edema, leukocyte adhesion and migration, production of cytokines, [50], [51] and the expression of adhesion molecules [47], [52]. In the present study, RAI with ATL increased the production of HO-1 in the pancreas, reduced the expression of adhesion molecules, such as ICAM-1 and PECAM-1, and decreased the production of TNF-α, IL-1β and IL-6, altogether strongly emphasizing the anti-inflammatory and pro-resolution effects induced by ATL through induction of HO-1 expression.

NF-κB is a nuclear transcription factor that plays an important role in the inflammatory process in acute pancreatitis. Activated NF-κB can integrate with the corresponding sequences in the gene promoters of a variety of inflammatory cytokines and adhesion molecules, and thus influence gene transcription, i.e. key factors involved in the pathological process of SIRS and MODS [53]. Being a central pro-inflammatory signalling regulator, the activation of NF-κB may initiate a pro-inflammatory ‘cytokine storm’ [54]. The activation of NF-κB is reduced by ATL [17], [55], and thus significantly influence on cytokine generation and adhesion molecules expression, confirmed by decreased levels of nuclear NF-κB p65, serum levels of TNF-α, IL-1β and IL-6 and the expression of ICAM-1 and PECAM-1 after RAI with ATL in the present study. The simultaneous decrease in cytokines and adhesion molecules indicate that ATL strongly decrease inflammatory mediator generation related to the NF-κB pathway. A previous study reported that the HO-1 gene promoter region contained a variety of regulatory elements, including the DNA binding sites of transcription factor NF-κB [56], suggesting that it plays a role in the regulation of HO-1 expression. Others have also confirmed that increased expression of HO-1 inhibit NF-κB activation [48], [57], [58], and the nuclear protein of NF-κB p65 increased notedly when blocking the induction of HO-1 [58]. We can conclude that the anti-inflammatory effects of HO-1 can be achieved by regulating the NF-κB signal pathway. RAI with ATL thus seem to attenuate the severity of SAP and the inflammatory response via induction of the expression of HO-1, down-regulating the activation of NF-κB, thereby reducing the generation of TNF-α, IL-1β and IL-6 and decreasing the expression of ICAM-1 and PECAM-1.

In conclusion, our study showed that CRAI with ATL not only attenuated the local pancreatitis process, but also down-regulated the systemic inflammatory response in experimental SAP rats. Moreover, our findings suggest that the potential mechanisms involve increased expression of HO-1, inhibiting the NF-κB signaling pathway, overall attenuating the severity of SAP, and SAP-associated SIRS.
